# Dr. Guy on the Influence of Employments upon Health

**Published:** 1844-03-09

**Authors:** 


DR. GUY ON THE INFLUENCE OF EMPLOYMENTS UPON HEALTH.

We gave, in our Number for November 18, a condensed account of the conclusions deducible from a former paper by Dr. Guy. He has since contributed another paper to the Journal of the Statistical Society, from which we take the following abstract:—

"A comparison of in-door with out-door occupations leads to the following results:—

"1. the ratio of cases of pulmonary consumption to those of other diseases is somewhat higher in persons following in-door occupations, than in those working in the open air; and this rule applies to both sexes.

"2. Pulmonary consumption occurs at an earlier age in men following in-door occupations than in those following out-door occupations.

"3. The probable excess of cases of pulmonary consumption in men following in-door occupations (for a higher ratio of consumptive cases in these employments is merely a strong presumption in favor of such excess), and the earlier age at which consumption makes its attack, would naturally tend to fill this class of employments with a greater number of young men, as well as to occasion a higher mortality at the early periods of life, and a lower average age of death. Accordingly, among those employed within doors, there is an excess of young persons, a higher mortality in early life, and a lower average age of death. The greatest age, as it happens, is the in the two classes.

"The classification of in-door employments, according to the amount of exertion which they require, leads to the following results:—

"1. The ratio of cases of pulmonary consumption to those of all other diseases is highest where the amount of exertion is least, and lowest where it is greatest; and the intermediate degree of exertion presents an intermediate ratio.

"2. The age at which pulmonary consumption makes its attack is earlier in employments requiring little exertion, than in those requiring more exertion, and in those requiring moderate exertion than in those demanding great effort.

"3. The per centage proportions of men under forty years of age following these three classes of employments is in strict accordance with the ratio of consumptive cases, and the ages at which consumption makes its attack; those proportions following the exact order of the degree of exertion.

"4. The age at death also follows the same order, the per centage proportion of deaths under forty being highest where there is least exertion, lowest where the exertion is greatest, and intermediate where there is a medium degree of exertion.

"5. The average age of death, also, is lowest where there is least exertion, but highest where the exertion is intermediate between the two extremes. The somewhat lower average obtained in the case of employments requiring great exertion, appears to depend on an excessive mortality under twenty years of age. The greatest age also occurs in occupations requiring a medium degree of exertion, the least maximum in those demanding the greatest effort.

"6. In the class of in-door occupations, with varied excercise (a class including the footman, waiter, &c.), the ratio of cases of pulmonary consumption ranks next to that of the sedentary occupations, and the per centage proportion of consumptive cases occurring before forty, the per centage proportion of men so employed under that age, and the per centage proporportion of deaths are higher than in any of the other classes, while the average age of death is lower. This class, however, stands alone, inasmuch as young men are in comparatively greater request, and old men comparatively little wanted.

"7. The class of out-door occupations requiring moderate exertion, presents a higher per centage proportion of deaths under forty, and a corresponding excess of young men; but the ratio of consumptive cases, and the per centage proportion of such cases occurring under forty are lower than in the class requiring greater exertion. This apparent anomaly may probably be explained by the fact that the attack of consumption is postponed till a later age in men following out-door employments, than in those working within doors.

"8. The maximum age in the case of men following the more laborious out-door employments is lower by one year than in those using less exertion, and in the latter there is a considerable excess of aged men.

"9. Sedentary employments, and those requiring little exertion, are more unfavorable to adult and middle age, but more favorable to old age, than those requiring greater efforts; on the other hand, employments requiring greater exertion are unfavorable to youth and longevity, but favorable to middle age. Employments requiring little exertion prove fatal, by inducing an excess of cases of pulmonary consumption early in life; those requiring great exertion, by occasioning other diseases of the air-passages and lungs, towards the commencement of old age.

"The following observations apply to certain occupations examined separately in the former essay, and to the effects of intemperance:—

"1. The exposure to a high temperature does not appear to exercise any injurious influence upon health during the early periods of life; but, in common with employments requiring a great amount of exertion, it is unfavorable to longevity.

"2. The inhalation of dust does not appear to be attended with the extremely injurious consequences which the high ratio of cases of pulmonary consumption would lead us to expect; but, when compared with the aggregate of other out-door occupations, the employment of the mason is found to be in some degree less favorable to health and longevity.

"3. Habits of intemperance appear to exercise a most injurious influence upon health; for men peculiarly exposed to the temptation of drinking present a high ratio of cases of consumption, a high per centage proportion of such cases occurring under forty, an excess of young men, an excess of deaths under forty, and especially between thirty and forty years of age, and a low average and maximum age.

"Lastly. A comparison of the age at death of gentlemen (including professional men), tradesmen, and artizans, issues in displaying the great advantage which the first class possesses over the other two, and the comparatively small difference which exists between the tradesman and the artizan. The average age of death of the first class exceeds that of the other two by ten years, while the average age of the tradesman exceeds that of the artizan only by a small portion of a year.

"The present inquiry, then, confirms the results to which other observers have arrived, by showing the great and undue advantage which the higher classes enjoy over other members of society. They live longer, and may be fairly presumed, while they live, to enjoy better health. This advantage is not to be wondered at, considering the better supply of air and food which they receive, the more spacious houses which they live in, and the greater care bestowed on the places which they inhabit. It is also partly due to the facilities which they enjoy for exercise in the open air. The great difference which exists between the gentry and other members of society, however, is not more worthy of notice than the very slight advantage which the tradesman enjoys over the class of working men. If we limit the comparison to the average age, we find that the tradesman lives about three-quarters of a year longer than the entire class of working men, little more than a year and a half longer than the class employed solely within doors, two years longer than men following the more sedentary occupations, and about three years longer than the class which consists chiefly of domestic servants. On the other hand, the life of the tradesman is shorter by about a quarter of a year than that of the entire class of out-door laborers.

"The little advantage possessed by the tradesman over the mass of working men probably results from the sedentary life he leads, his want of proper exercise, and the small space which the necessities of business allow him to give to the accommodation of himself and family. Taking one tradesman with another, it is, perhaps, not unreasonable to suppose that he habitually breathes an air as impure, and follows an occupation nearly as unwholesome, as the class beneath him. The labour which the working man has to undergo, provided it is not carried to excess, is more favorable to health than the confinement to which the tradesman is subject, and this confinement raises him in the scale of mortality but little above men following the more sedentary occupations. Both classes of men—the tradesman and the working man—are doubtless exposed to unwholesome influences, which might be wholly removed or greatly mitigated by the interference of others, or by their own precautions. Both classes probably suffer to a greater degree than they imagine from those habits of intemperance from which the higher classes have nearly emancipated themselves, but which still form the great physical and moral bane of the mass of our population.

"The bad effects of sedentary habits, on the one hand, and of laborious exertion on the other, have been fully demonstrated in the course of this and the former essay. How much of the waste of life and health is due to the bad air which the in-door laborer is constrained to breathe; how much to the inclemencies of the weather to which the out-door laborer is exposed; how much to the unhealthy habitations which both classes are compelled to live in; and how much to the bad habits in which both indulge, it is impossible to determine. These causes are so mixed up together as not to admit of that separation which must precede a correct estimate of their comparative influence. But that unwholesome dwellings, and bad air, and intemperate habits do tend to injure health and shorten life, there is abundant proof; that the permission of such things, where it is possible to rectify or prevent them, is bad economy, there can be no doubt. Unfortunately for the sufferers by these unwholesome influences, it is much more easy to establish general principles concerning them than to point out the precise amount of injury which they occasion. How many thousand unnecessary victims pulmonary consumption claims year by year it is impossible to ascertain; whether this chronic plague is more or less destructive than the kindred pestilence which is constantly snatching away so many of our adult population must remain a subject of conjecture; but of this there can be no reasonable doubt, that in addition to those who might have escaped consumption, and reached at least the borders of old age, a large proportion of all who die of that disease die much earlier than they would if they had been placed in more favorable circumstances. As each unnecessary death from consumption represents the loss, after a slow and lingering illness, of one of those who form the real strength of society, so the death of each father of a family at an age when those who depend upon him for support are most helpless, is the source of an amount of private suffering and privation, and oftentimes of expense to the public, which is much more easily conceived than estimated. To complete the calculation of the cost to society in this one disease alone, which results from unwholesome influences admitting of removal, it would be necessary to add the hereditary taint transmitted from the consumptive parent, and ready at a fitting season to cut short the life of a child after he in his turn has bequeathed the same sad legacy to his offspring. Thus it happens that the seeds of disease are more thickly sown with each new generation, and death reaps an earlier and more abundant harvest, and a race of men famed for strength and vigor is doomed to slow but sure degeneracy."

*Prov. Med. and Surg. Journ.* Dec. 16, 1843.

